# Latest Novelties on Plasmonic and Non-Plasmonic Nanomaterials for SERS Sensing

**DOI:** 10.3390/nano10061200

**Published:** 2020-06-19

**Authors:** Grégory Barbillon

**Affiliations:** EPF-Ecole d’Ingénieurs, 3 bis rue Lakanal, 92330 Sceaux, France; gregory.barbillon@epf.fr

**Keywords:** SERS, sensors, plasmonics, zinc oxide, metal oxides, self-assembly, bimetallic nanoparticles

## Abstract

An explosion in the production of substrates for surface enhanced Raman scattering (SERS) has occurred using novel designs of plasmonic nanostructures (e.g., nanoparticle self-assembly), new plasmonic materials such as bimetallic nanomaterials (e.g., Au/Ag) and hybrid nanomaterials (e.g., metal/semiconductor), and new non-plasmonic nanomaterials. The novel plasmonic nanomaterials can enable a better charge transfer or a better confinement of the electric field inducing a SERS enhancement by adjusting, for instance, the size, shape, spatial organization, nanoparticle self-assembly, and nature of nanomaterials. The new non-plasmonic nanomaterials can favor a better charge transfer caused by atom defects, thus inducing a SERS enhancement. In last two years (2019–2020), great insights in the fields of design of plasmonic nanosystems based on the nanoparticle self-assembly and new plasmonic and non-plasmonic nanomaterials were realized. This mini-review is focused on the nanoparticle self-assembly, bimetallic nanoparticles, nanomaterials based on metal-zinc oxide, and other nanomaterials based on metal oxides and metal oxide-metal for SERS sensing.

## 1. Introduction

The strong development of plasmonic nanomaterials for various applications such as photovoltaics [1,2,3,4], optical devices [5,6,7,8,9,10], and biochemical sensors [11,12,13,14,15,16,17] has taken place over these last ten years. The plasmonic nanostructures can also enable the detection of phase transitions under high-pressure conditions [18], the luminescence upconversion enhancement [19,20], and the optical tuning of photoluminescence [21] and upconversion luminescence [22]. For plasmonic sensors of biomolecules, the surface enhanced Raman scattering (SERS) is largely employed as a very sensitive technique of analysis. For maximizing the enhancement factor (EF) of SERS signal, the electromagnetic contribution is predominantly used. EF is calculated by taking the fourth power of the electric field amplitude obtained with the plasmonic nanostructures [23]. The key point in order to obtain zones of strong electric field (called hotspots) is a precise control of the shape, size, and spatial organization of plasmonic nanostructures. The control of these parameters is enabled and realized thanks to a great number of lithographies such electron beam lithography [24,25,26,27], optical lithographies [28,29,30], nanosphere lithography [31,32,33], and nanoimprint lithography [34,35,36]. Several groups examined a broad number of designs as plasmonic nanodisks, nanodimers, and nanorods, which have reached important EF values (EF = 10^6^–10^9^) [37,38,39]. Furthermore, a gain of 1 or 2 orders of magnitude on the enhancement factor can be realized by inserting a metallic layer under the plasmonic nanosystems (EF = 10^6^–10^9^). A coupling between the nanosystems by means of surface plasmon polaritons on the metallic film [40,41] or hybridization of localized plasmon modes with the image modes in a plasmonic substrate [42,43] allows this gain. Moreover, this advanced type of SERS substrate can allow an application to multispectral SERS sensing [44]. Another approach for obtaining an excellent SERS activity is to use luminescent-plasmonic material based on neodymium(III)-doped yttrium–aluminium–silicate microspheres with gold nanoparticles [45]. In addition, hybrid metal/Si nanostructures allowed achieving substantial values of EF (10^7^–10^10^) [46,47,48,49,50,51,52]. These hybrid nanostructures based on silicon (semiconductor) have the property of biocompatibility, and a low cost of production. Moreover, they permit the emergence of hotspots placed at the level of the interface of the metal and semiconductor. Furthermore, another possible outcome is based on the zinc oxide (ZnO) nanostructures capped with metallic layer or metallic nanoparticles in order to achieve excellent enhancement factors (EF = 10^6^–10^10^) [53,54,55,56]. The use of bimetallic nanosystems offers the possibility to have excellent functionalities concerning the plasmonic and chemical properties compared to plasmonic nanosystems composed of an unique metal [57,58]. As silver has a better plasmonic enhancement than gold, the bimetallic gold-silver nanosystems are developed in order to suppress oxidation of silver with gold [59]. Thus, sharper and stronger characteristics of localized surface plasmon resonances (LSPRs) for the bimetallic systems enable obtaining larger SERS activities due to the hotspots coming from the LSPR coupling between Au and Ag nanosystems [60,61]. Another way is to design effective SERS substrates by self-assembling of plasmonic nanoparticles [62,63]. The advantages of the self-assembly are the low cost and time of fabrication of SERS substrates. This effectiveness is strongly depending on the distance between plasmonic nanoparticles [64]. Nevertheless, the reproducibility of the SERS signal is very weak with this type of substrates [65]. However, improvements have emerged as the template-assisted self-assembly suppressing the issue of signal reproducibility [66,67]. In addition, alternative materials such as metal oxides (different of zinc oxide as MoO_3_ molybdenum trioxide, WO_3−*x*_ tungsten oxide or CoFe_2_O_4_ cobalt ferrite) emerged for SERS application [68,69].

The aim of this mini-review is to present the latest novelties on plasmonic and non-plasmonic nanomaterials for SERS sensing over the period 2019–2020. We will focus on the self-assembly of plasmonic nanoparticles in a first part. Then, bimetallic nanosystems will be addressed, then nanomaterials based on metal-ZnO, and finally other nanomaterials based on metal oxides and metal oxide-metal.

## 2. Novelties on Plasmonic and Non-Plasmonic Nanomaterials for SERS Sensing

In order to compare the different SERS performances for all the nanosystems presented in this mini-review, the detection limits (LODs) obtained experimentally were used, and also the calculation of the enhancement factor (EF) [37] or the analytical enhancement factor (AEF) [39] (see tables of each section). The formulas of EF and AEF were expressed as follows:

(1) $$\begin{array}{c} EF=\frac{I_{SERS}}{I_{Raman}}\times \frac{N_{Raman}}{N_{SERS}} \end{array}$$

(2) $$\begin{array}{c} AEF=\frac{I_{SERS}}{I_{Raman}}\times \frac{C_{Raman}}{C_{SERS}} \end{array}$$

where $I_{SERS}$, $I_{Raman}$ represent the SERS and Raman intensities, respectively. $N_{SERS}$, $N_{Raman}$, $C_{SERS}$, $C_{Raman}$ are the numbers and concentrations of analyte molecules for SERS and reference Raman experiments, respectively.

### 2.1. SERS Substrates Designed by Self-Assembly

Novel SERS substrates were designed by self-assembly in the period 2019–2020 (see Table 1). The first example concerns the fabrication of nanogap plasmonic micropillars by using the capillary-force driven self-assembly (CFSA). These SERS substrates allowed achieving enhancement factors up to 8 × 10^7^ in a fluidic medium. Moreover, a detection limit (LOD) of 0.1 mM for doxorubicin (DOX = anticancer drug) was reached with this type of structures. This fabrication method was very flexible, because it allowed realizing plasmonic structures on flat and non-flat substrates [70]. Ghosh et al. showed that plasmonic dimers with subnanometer gap enabled to reach enhancement factor of 10^7^ and a Rhodamine 6G (R6G) detection at the ppb level. These plasmonic nanostructures were realized by directed microwave-assisted self-assembly and segregated by a graphene monolayer [71].

**Figure 1:**
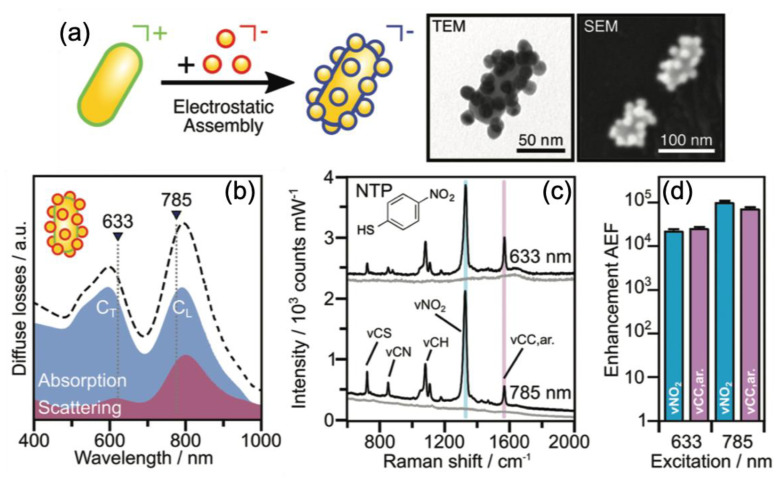
(**a**) Scheme of the self-assembly of Au nanospheres on Au nanorods. TEM and SEM images of the obtained superstructures. (**b**) Absorption and scattering spectra of the plasmonic superstructures with the chosen wavelengths of excitation for the transversal (C_*T*_) and longitudinal (C_*L*_) coupled modes, which are 633 nm and 785 nm, respectively. (**c**) SERS spectra of plasmonic superstructures without (in grey) and with 4-NTP molecules (C_*NTP*_ = 1 μM, in black) for the two wavelengths of excitation. (**d**) Analytical enhancement factor (AEF) values corresponding to the SERS spectra in (**c**). All the figures are reproduced from [72] with permission from the Royal Society of Chemistry.

Kuttner et al. reported on SERS performances obtained with gold nanorods on which were deposited self-assembled Au nanospheres (see Figure 1). High AEF values of 10^4^–10^5^ were achieved due to the coupling of plasmonic modes of gold nanorods and gold nanospheres (see Figure 1). The 4-nitrothiophenol (4-NTP) molecules were used in order to determine the AEF value [72]. From Figure 1, it has been also observed that the AEF values were higher of a magnitude order for the longitudinal (C_*L*_) coupled mode than the transversal (C_*T*_) coupled mode. This was due to the fact that the excitation wavelength had a better position in comparison to the resonance position of the C_*L*_ mode [72].

**Figure 2:**
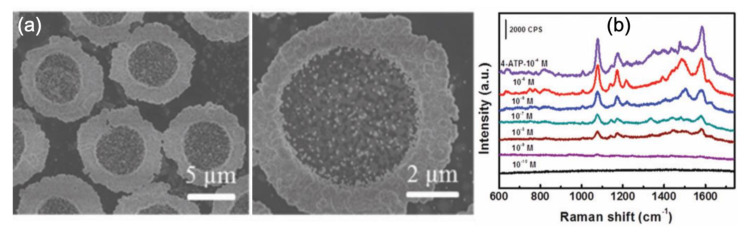
(**a**) SEM images of the obtained Pt disks decorated by gold micro-rings. (**b**) SERS spectra of 4-ATP molecules for different concentrations. All the figures are reproduced from [75] with permission from the Royal Society of Chemistry.

Fusco et al. discussed SERS performances of gold nano-island (NI) substrates obtained by self-assembly of which the disorder degree of NIs was controllable. The reached EF values were in the range of 10^7^–10^8^ and the lowest concentration detected was 1 nM with the R6G molecules [73]. Wu et al. showed an one-step method of fabrication of SERS substrates constituted of gold nanoparticles (AuNPs) and a polyvinyl chloride (PVC) film via an interfacial self-assembly induced by polymer. This fabrication of these AuNPs/PVC films was simple, low cost and these films can be reused. Moreover, the SERS performances were excellent such as an EF of 3.7 × 10^6^ for the sensing of 4-aminothiophenol (4-ATP) molecules, and a LOD of 10 ng·cm^−2^ for pesticides (thiram) [74]. To conclude this section, Yin et al. fabricated bimetallic arrays composed of gold micro-rings, which decorated platinum (Pt) disks (see Figure 2) by employing a process of templated-self-assembly (TSA). Thus, a SERS enhancement of 4.2 × 10^5^ was attained for the sensing of 4-aminothiophenol molecules (4-ATP). The limit of detection of 4-ATP obtained with these superstructures was equal to the concentration of 1 nM (see Figure 2) [75].

### 2.2. Bimetallic Nanoparticles for SERS Sensing

Recent advances on bimetallic nanoparticles for SERS sensing have occurred over the period of 2019–2020 (see Table 2). Firstly, Su et al. reached an improved sensitivity of detection of cardiorenal syndrome markers by using the combination of 3D ordered macroporous Au-Ag-Au array (substrate) and Ag-Au nanostars (nanotags). Thus, this combination enabled generating hotspots coming from the plasmonic coupling in near-field inducing a SERS enhancement. The LODs observed experimentally with this plasmonic nanosystem (substrate+nanotags) were 0.41, 0.53, and 0.76 fg·mL^−1^ for neutrophil gelatinase-associated lipocalin (NGAL), N-terminal prohormone of brain natriuretic peptide (NT-ProBNP), and cardiac troponin (cTnI), respectively [76]. Ning et al. also demonstrated SERS performances obtained by using Au–Ag–Ag nanorod coupled to the magnetic beads through DNA hybridization. A LOD of 1 fM for HPV-16 fragments (human papillomavirus DNA type 16) was found [77]. Hussain et al. reported on the quick contaminant detection in milk by using Au/Ag core-shell nanoparticles. LODs of 0.21 and 14.88 ppm for thiram and dicyandiamide (DCD) were obtained in milk, respectively. The detection limits of molecules of interest with these plasmonic Au/Ag nanoparticles can be thus determined in a short time (34 min) by using the fabrication approach proposed in this paper [78]. Tian et al. showed the easy synthesis of Au/Ag nanoparticles rich in silver by using the combination of galvanic replacement process and co-reduction of silver atoms. These bimetallic nanoparticles enabled obtaining an improved SERS activity caused by the great presence of silver in the nanoparticles. In order to evaluate the SERS activity of these Au/Ag nanoparticles rich in silver, the authors chose using thiophenol molecules, and an EF value of 2.3 × 10^6^ was found. Then, the authors labeled their Au/Ag nanoparticles with Atto-610 antibodies and added gold nanoparticles through electrostatic adsorption for the SERS detection of rabbit IgG. Thus, the authors achieved a LOD of 20 pg.L^−1^ for rabbit IgG [79].

William et al. demonstrated SERS performances of sprouted potato-shaped bimetallic nanoparticles. The shape of these Au/Ag nanoparticles was obtained by carefully setting the quantity of silver for a given quantity of gold. Thus, the authors obtained a LOD of 1 fM for methylene blue molecules [80]. Joseph et al. reported on the fabrication AuAg@Ag hollow cubic nanosystems for a detection of mercaptothiophenol (4-MPh) molecules. These structures were composed of an AuAg core and an Ag shell. A LOD of 1 aM was found for 4-MPh molecules. This efficiency of these SERS nanostructures was caused by the electromagnetic and chemical contributions. The major part of this efficiency was due to the charge transfer of 4-MPh molecules via the silver shell to the alloy core [81]. Vu et al. realized Ag-Au nanostructures on nickel foam as SERS substrates. Authors employed rhodamine 6G molecules in order to test their 3D nanostructures, and a LOD of 0.1 nM was found for these molecules. Moreover, the SERS signal was durable even after 100 cycles of abrasion with sandpaper, or after sonication for half an hour for these Ag-Au nanostructures on nickel foam [82]. Yilmaz et al. demonstrated a SERS activity with bimetallic core–shell nanoparticles with an intermediate layer of bioinspired polydopamine between Au and Ag nanoparticles. An EF value of 3.5 × 10^5^ for the detection of methylene blue molecules was found. This bioinspired polydopamine layer is employed as stabilizing agent for adsorption of silver nanoparticles as well as reducing agent for reduction of Ag ions [83].

**Figure 3:**
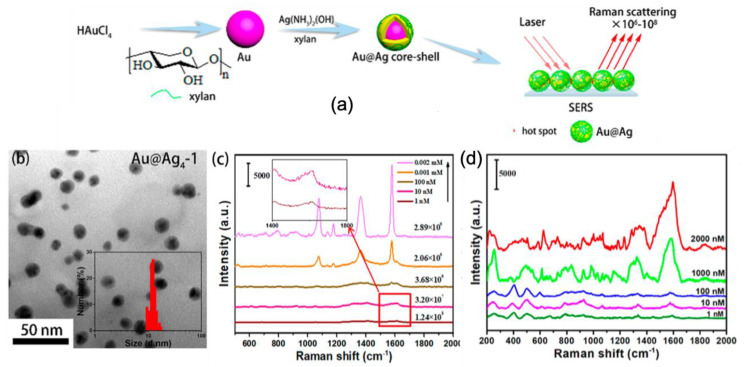
(**a**) Principle scheme of the synthesis of Au/Ag core-shell nanoparticles by using xylan for SERS sensing. (**b**) TEM image of xylan-capped Au@Ag nanoparticles with optimized parameters for SERS. SERS spectra of (**c**) 4-MBA molecules and (**d**) Sudan I molecules for different concentrations obtained with optimized xylan-capped Au/Ag nanoparticles of (**b**). All the figures are reprinted (adapted) with permission from [84], Copyright 2019 American Chemical Society.

Furthermore, Cai et al. demonstrated the green synthesis Au–Ag core-shell nanoparticles by using xylan for SERS sensing (see Figure 3). With these xylan-capped Au@Ag nanoparticles, a detection limit of 1 nM and an AEF value of 1.24 × 10^8^ for 4-mercaptobenzoic acid (4-MBA) molecules were found as well as a LOD of 1 nM for Sudan I molecules (food contaminant; see Figure 3). These SERS performances were explained by the fact that the xylan capping allowed creating hotspots between Au/Ag nanoparticles. Optimal SERS performances for xylan-capped Au/Ag nanoparticles were realized for a mole ratio of AgNO_3_ to HAuCl_4_ equal to 4 and a dosage of xylan equal to 1 in the fabrication of Ag shell corresponding to 2.86 × 10^−8^ mol of xylan (see Figure 3) [84].

**Figure 4:**
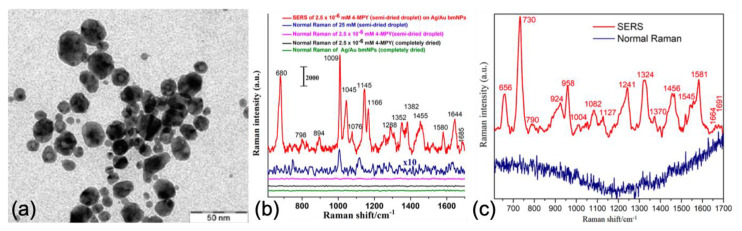
(**a**) TEM image of Au/Ag nanoparticles. (**b**) SERS spectrum of 4-MPY molecules at 2.5 nM concentration (in red). (**c**) SERS spectrum of *Escherichia coli*. All the figures are reprinted (adapted) with permission from [85], Copyright 2020 American Chemical Society.

To conclude this section on bimetallic nanosystems, Prakash et al. reported on the SERS detection of bacteria by using Au/Ag plasmonic nanoparticles which were positively charged (see Figure 4). Firstly, authors tested the SERS properties of these plasmonic nanoparticles by employing mercaptopyridine (4-MPY) molecules. An AEF of 3.5 × 10^7^ and a LOD inferior to nanomolar concentration were found (see Figure 4). Then, the detection of bacteria (e.g., *Escherichia coli*) was realized as proof-of-concept (see Figure 4). Thus, these bimetallic nanoparticles charged positively allowed basic experimental conditions without using specific processes for SERS sensing of bacteria [85].

### 2.3. Nanomaterials Based on Metal-ZnO for SERS Sensing

**Figure 5:**
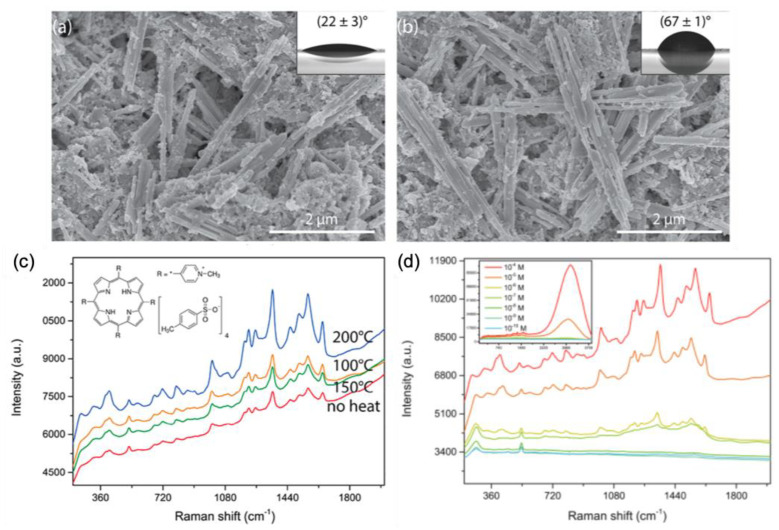
SEM pictures of (**a**) ZnO nanowires not annealed, and (**b**) annealed at 200 °C. Insets display contact angles for each surface. (**c**) SERS spectra of TMPyP recorded on Ag/ZnO nanowires for several temperatures. (**d**) SERS spectra of TMPyP recorded on Ag/ZnO nanowires annealed at 200 °C for various concentrations of TMPyP. Inset in (**d**) displays the whole spectrum with fluorescence signal that appears when the concentration is superior or equal to 10^−5^ M. All the figures are reprinted (adapted) with permission from [86], Copyright 2020 American Chemical Society.

The use of zinc oxide associated to a noble metal for the fabrication of highly efficient SERS substrates increased since these last years. In this section, we present latest works on this subject over the period 2019–2020 (see Table 3). Fularz et al. reported on SERS performances of ZnO nanowires coated with Ag nanoparticles. Their idea was to treat these hybrid nanostructures by heat, which enabled an efficient charge transfer in order to enhance the SERS signal. This heat processing in oxygen environment introduced defects as interstitial oxygen in ZnO structure. This interstitial oxygen reduced optical gap favoring the charge transfer between hybrid nanowires and molecules. Moreover, the heat processing changed the wettability of Ag/ZnO nanowires (see Figure 5a,b) that had for effect of decreasing the spreading of Ag nanoparticles or studied molecules on ZnO nanowire surface which also favored the SERS enhancement. Figure 5c displays the effect of the annealing temperature of Ag/ZnO nanowires on the SERS spectra of (meso-tetra(N-methyl-4-pyridyl)porphine tetrachloride (TMPyP) molecules. A LOD of 100 nM is found for TMPyP molecules with Ag/ZnO nanowires annealed at 200 °C (see Figure 5d) [86].

**Figure 6:**
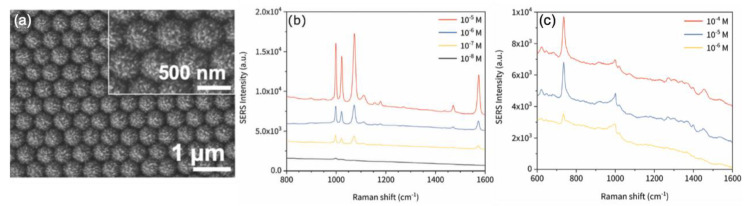
(**a**) SEM picture of Au/ZnO nano-urchins. SERS spectra of thiophenol (**b**) and adenine (**c**) molecules recorded on Au/ZnO nano-urchins for several concentrations of the studied molecules. All the figures are reproduced from [95] with permission from the Royal Society of Chemistry.

The following examples present two studies reporting on the SERS performances of Ag/ZnO heterostructures. Firstly, Yao et al. investigated SERS performances of Mg-doped ZnO heterostructures coated with Ag nanoparticles. Authors demonstrated that the enhancement of SERS signal was due to the combination of electromagnetic contribution and charge transfer, and they reported a LOD of 0.1 pM for detection of malachite green molecules [87]. Secondly, Rajkumar and Sarma demonstrated excellent SERS performances obtained with Ag/ZnO heterostructures composed of ZnO microrods decorated by Ag nanoparticles. This design allowed obtaining a clustering of Ag nanoparticles inducing the formation hotspots. These hotspots enabled the enhancement of the SERS signal. Thus, LOD of 1 pM and AEF of 1.3 × 10^10^ were found with these heterostructures for the detection of methylene orange molecules [88]. Next, Zhu et al. reported on the use of a hybrid microcavity composed of ZnO, graphene and silver for enhancing the SERS signal. This enhancement is obtained by combining a whispering-gallery mode of a microcavity, the plasmonic resonance of Ag nanoparticles and a charge transfer between studied molecules and graphene. Thus, an EF value of 9.5 × 10^11^ and a LOD of 1 fM were obtained with this structure for detection of rhodamine 6G molecules [89]. Wang et al. investigated SERS performances of hollow ZnO@Ag nanospheres for detection of nitrite species. Authors demonstrated a LOD of 3 nM for detection of nitrite species [90]. In the next examples, four studies based on metal/ZnO nanorods are presented. The first one concerns Ag/ZnO nanorods. In this first investigation, authors demonstrated SERS performances due to charge transfers. LODs of 1 nM and 5 nM were found for detection of pioglitazone and phenformin, respectively [91]. Then, Pal et al. demonstrated that a bimetallic/ZnO structure composed of silver, zinc oxide, and gold allowed significant SERS performances. Authors reported an excellent LOD of 0.3 nM for detection of lambda DNA [92]. The last two examples are dedicated to the SERS performances of Au/ZnO nanorods. At first, Zhou et al. showed that Au/ZnO heterogeneous nanorods allowed an enhancement of SERS signal due to charge transfer enhanced by the localized surface plasmon resonance of a gold nanoparticle located at an extremity of ZnO nanorods. A value of the enhancement factor of 1.2 × 10^4^ was determined for detection of dopamine [93]. Next, Doan et al. reported on the use ZnO nanorods coated with gold nanoparticles for enhancing SERS performances. Authors also demonstrated that their SERS substrates were self-cleaning under UV light, and found a LOD of 1 nM for detection of methylene blue molecules [94]. To finish this section on metal-ZnO-based nanomaterials, Graniel et al. reported on the fabrication of Au/ZnO hollow nano-urchins (see Figure 6a) and their SERS performances. These hybrid nano-urchins enabled the formation of hotspots (strong electric field zones) which induced enhancements of SERS signal. Thus, authors found LODs of 10 nM and 1 μM for detection of thiophenol and adenine, respectively (see Figure 6b,c). Moreover, they demonstrated an excellent substrate-to-substrate reproducibility with a relative standard deviation < 10% [95].

### 2.4. Nanomaterials Based on Metal Oxides and Based on Metal Oxide-Metal for SERS Sensing

In the previous section, we discussed the nanomaterials based on zinc oxide. In this section, recent novelties concerning to other nanomaterials based on metal oxides, then based on metal oxide-metal are presented for SERS sensing on the period of 2019–2020 (see Table 4).

**Figure 7:**
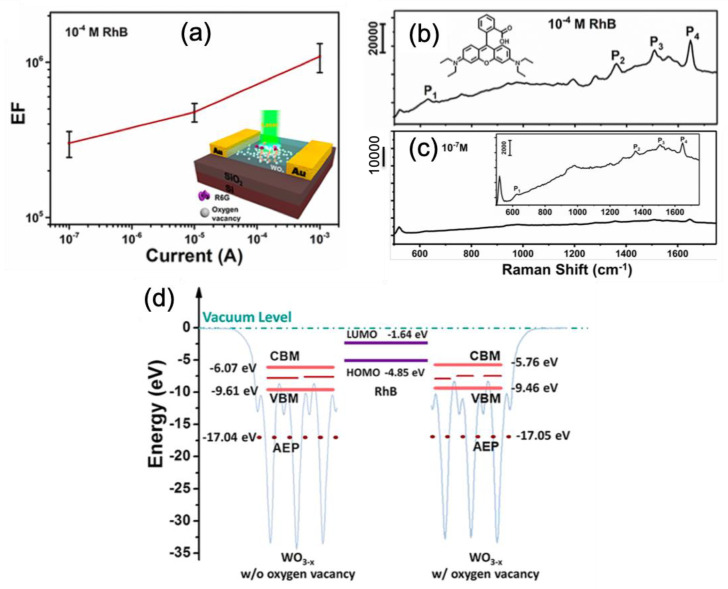
(**a**) Enhancement factor for SERS signal versus leakage current for a rhodamine B (RhB) concentration of 0.1 mM, and the inset represents the structure scheme. SERS spectra of RhB recorded for a leakage current of 1 mA with an RhB concentration of (**b**) 0.1 mM and (**c**) 0.1 μM (at detection limit (LOD)), and the insets of the figures (**b**) and (**c**) represent a molecular scheme of RhB and a zoom on the SERS spectrum, respectively. (**d**) Illustration and alignment of energy levels of WO_3−*x*_ film without oxygen vacancies (at left), RhB molecule (at center), and WO_3−*x*_ film with oxygen vacancies (at right). All the figures are reprinted (adapted) with permission from [99], Copyright 2019 American Chemical Society.

In the first four examples, nanomaterials based on metal oxides are presented for SERS sensing. He et al. reported on the SERS performances of few-layered MoO_3_ nanosheets deposited on a SiO_2_/Si substrate. Authors demonstrated that SERS enhancement was due to a chemical mechanism when the thickness of these nanosheets was reduced. Moreover, this chemical mechanism was further enhanced via an atomic intercalation in the van der Walls gap. Thus, a LOD of 20 nM for detection of rhodamine 6G molecules was obtained with these MoO_3_ nanosheets [96]. Hou et al. proposed an alternative strategy to classical plasmonic nanostructures for the fabrication of efficient SERS substrates. This strategy was to use non-stoichiometric transition metal oxides (TMOs) as SERS substrates. These planar TMOs SERS substrates were realized through a magnetron sputtering coupled to H_2_ annealing. In this study, authors chose to investigate the following non-stoichiometric groups of TMOs: IVB, VB and VIB. They obtained a lowest LOD of 1 nM for detection of rhodamine 6G molecules. The SERS enhancement was due to the mechanism of photoinduced charge transfer from oxygen vacancies [97]. Wang et al. demonstrated the SERS performances of a W_18_O_49_-H_2_ nanowire film. A LOD of 0.1 μM and an EF value of 4.4 × 10^5^ were found for detection of rhodamine B (RhB) molecules. These SERS performances were due to the presence of oxygen vacancies in W_18_O_49_-H_2_ film of which the vacancy number was increased by the reduction of H_2_. This improved number of oxygen vacancies enabled enriching surface states of substrate allowing the adsorption of an increased number of RhB on this same substrate and thus inducing the enhancement of SERS activity through a charge transfer mechanism [98]. Zhou et al. demonstrated an electrical control of SERS enhancement based on tungsten oxide surface (WO_3−*x*_) deposited on a SiO_2_/Si substrate. This SERS improvement was realized by an electric field that introduced defects (oxygen vacancies) in the tungsten oxide surface (see the structure scheme in Figure 7a) resulting in a better charge transfer between oxide surface and the studied molecules (here, rhodamine B = RhB). A LOD of 0.1 μM and an enhancement factor of 1.2 × 10^6^ were found for detection of rhodamine B molecules (see Figure 7b,c) with a programmed current of leakage equal to 1 mA. From Figure 7a, this value of 1 mA for the leakage current was optimal for enhancing the SERS signal. The EF value was equal to 1.2 × 10^6^ for this current. The SERS improvement was due to a charge transfer that was favored by good alignment of the energy levels of oxygen vacancies with the molecular energy levels of rhodamine B (see Figure 7d) [99].

In the last two examples, nanomaterials based on metal oxide-metal are presented for SERS sensing. In the first one, Wei et al. reported on the SERS performances of Ag nanowires decorated with WO_3−*x*_ quantum dots (WO_3−*x*_ QD/AgNW). The SERS activity was investigated by using methylene blue (MB) molecules (MB concentration used is 1 μM that was taken as the LOD here). Authors demonstrated that WO_3_ QD/AgNW films had a better SERS activity than WO_2.72_ QD/AgNW films when no irradiation with Xe lamp was applied. On the contrary, WO_2.72_ QD/AgNW films had a better SERS activity than WO_3_ QD/AgNW films when irradiation with Xe lamp was applied. Authors observed a decreasing of SERS activity when the content of WO_3_ QDs was increased, and stated that the localized surface plasmon resonance along AgNWs was blocked by the presence of WO_3_ QDs. Authors observed the same behavior with WO_2.72_ QD/AgNW films. Moreover, when WO_3_ QD/AgNW films were irradiated, the SERS activity was decreased due to the photo-decomposition of methylene blue molecules. However, a contrary effect was observed with WO_2.72_ QD/AgNW films. Indeed, the SERS activity was improved when the irradiation time was increased. This was due to the presence of oxygen defects in WO_2.72_ QDs which favored charge transfers (electrons) inducing the SERS enhancement [100].

**Figure 8:**
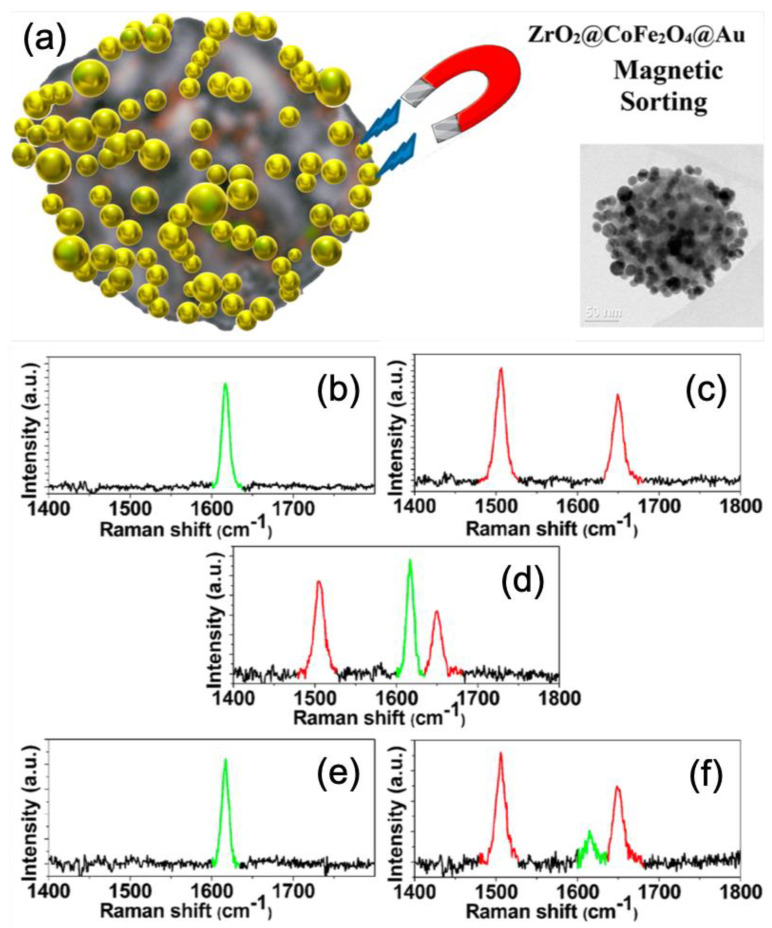
(**a**) Scheme and TEM picture of a magnetoplasmonic nanoparticle. SERS spectra of (**b**) malachite green on magnetoplasmonic NPs, (**c**) texas red on non-magnetic plasmonic NPs, (**d**) the mixture of (**b**) and (**c**) in solution. (**e**) SERS spectrum recorded after magnetic attraction from the mixed solution and re-dispersed in water. (**f**) SERS spectrum recorded after magnetic attraction with the mixed solution. All the figures are reprinted (adapted) with permission from [101], Copyright 2020 American Chemical Society.

In the last example concluding this section, Del Tedesco et al. reported on the use of magnetoplasmonic nanoparticles for enhancing the SERS signal and its separation effect of magnetic and non-magnetic systems. In this study, a magnetoplasmonic nanoparticle was composed of a mesoporous nanoparticle of ZrO_2_ on which cobalt ferrite (CoFe_2_O_4_) nanoparticles were deposited, then they were functionalized with gold nanoparticles (see Figure 8a). Other nanoparticles (NPs) were realized and only composed of a mesoporous nanoparticle of ZrO_2_ on which gold nanoparticles were deposited in order to investigate the separation effect of magnetic and non-magnetic systems. Authors calculated an enhancement factor of around 5 × 10^10^ for SERS signal with these magnetoplasmonic nanoparticles. Moreover, the magnetoplasmonic and non-magnetic plasmonic nanoparticles were functionalized with thiolated malachite green (MG) and thiolated texas red (TR), respectively (see Figure 8b,c). Thus, authors demonstrated the separation of magnetoplasmonic and non-magnetic plasmonic nanoparticles with magnetic sorting (see Figure 8d–f). Indeed, Figure 8d displays the SERS spectrum of the solution of the mixture of the MG-functionalized magnetoplasmonic (Raman peak in green) and TR-functionalized plasmonic nanoparticles (Raman peaks in red). The SERS spectrum displayed in Figure 8e corresponds to this recorded after magnetic attraction and re-dispersed in water. The last SERS spectrum shown in Figure 8f corresponds to this recorded with the starting solution (mixture of two types of NPs) after magnetic attraction, where it only remained that the red Raman peaks corresponding to non-magnetic plasmonic nanoparticles, and also with a small remaining SERS signal (in green) corresponding to MG-functionalized magnetoplasmonic nanoparticles [101].

## 3. Conclusions

In this short review, recent novelties on plasmonic and non-plasmonic nanomaterials for SERS sensing were summarized in four major parts: (i) self-assembly of plasmonic nanoparticles, (ii) bimetallic nanosystems, (iii) nanomaterials based on metal-zinc oxide, and (iv) nanomaterials based on metal oxides and metal oxide-metal. From these nanomaterials, excellent SERS performances have been obtained thanks to the generation of hotspots or an improved charge transfer. Thus, enhancement factors were in the range of 10^4^–10^8^, 10^5^–10^8^, 10^4^–10^12^, and 10^5^–10^10^ for part (i), (ii), (iii) and (iv), respectively. For LOD, the values were in the range of 1 nM–0.1 mM, 1 aM–1 nM, 1 fM–1 μM, and 1 nM–0.1 μM for part (i), (ii), (iii), and (iv), respectively. By taking into account these different values of EF and LOD, the best SERS nanomaterials are bimetallic nanosystems, and nanostructures based on metal-zinc oxide, even if other nanomaterials based on metal oxides and metal oxide-metal are also good potential candidates. Moreover, this type of the fabrication strategy and nanomaterials also allowed quick, low-cost, reproducible generation of efficient SERS substrates and SERS nanotags. However, the physical/chemical properties of SERS substrates must be optimized, as coupling of the molecules with plasmonic surface, preferably in hotspots, for instance. All these properties can be optimized by using numerical simulations and experimental measurements, which are essential for acquiring a deeper understanding of all key points and achieving an efficient transfer of SERS as a regular analytical technique in the near future.

## Figures and Tables

**Table 1:** Surface enhanced Raman scatterinSurface enhanced Raman scattering (SERS) performances of substrates designed by self-assembly for biological/chemical sensing (LOD = limit of detection; DOX = doxorubicin; 4-ATP = 4-aminothiophenol; PVC = polyvinyl chloride; 4-NTP = 4-nitrothiophenol).

SERS Substrates	Detected Molecules	EF or AEF	LOD	References
3D Au Nanogap micropillars	Rhodamine 6G	8 × 10^7^	–	[70]
3D Au Nanogap micropillars	DOX	–	0.1 mM	[70]
Au-Graphene-Au dimers	Rhodamine 6G	10^7^	ppb level	[71]
Au nanorods with Au spheres	4-NTP	10^4^–10^5^	–	[72]
Au nanoislands with disorder control	Rhodamine 6G	10^7^–10^8^	1 nM	[73]
AuNPs/PVC film	4-ATP	3.7 × 10^6^	–	[74]
AuNPs/PVC film	thiram	–	10 ng.cm^−2^	[74]
Bimetallic array with Au microrings	4-ATP	4.2 × 10^5^	1 nM	[75]

**Table 2:** SERS performances of bimetallic nanoparticles for biological/chemical sensing (4-MBA = 4-mercaptobenzoic acid; cTnI = cardiac troponin; NT-ProBNP = N-terminal prohormone of brain natriuretic peptide; NGAL = neutrophil gelatinase-associated lipocalin; HPV-16 = human papillomavirus DNA type 16; DCD = dicyandiamide; 4-MPY = Mercaptopyridine; MB = Methylene blue; 4-MPh = 4-mercaptothiophenol; PDOP = Polydopamine).

SERS Substrates	Detected Molecules	EF or AEF	LOD	References
3DOM Au-Ag-Au array with Ag-Au stars	cTnI	–	0.76 fg·mL^−1^	[76]
3DOM Au-Ag-Au array with Ag-Au stars	NT-ProBNP	–	0.53 fg·mL^−1^	[76]
3DOM Au-Ag-Au array with Ag-Au stars	NGAL	–	0.41 fg·mL^−1^	[76]
Au@AgAg nanorods	HPV-16	–	1 fM	[77]
Au@AgNPs	thiram	–	0.21 ppm	[78]
Au@AgNPs	DCD	–	14.88 ppm	[78]
Au@AgNPs	rabbit IgG	–	20 pg·L^−1^	[79]
Au@AgNPs	Thiophenol	2.3 × 10^6^	–	[79]
Potato shaped Au-Ag NPs	MB	–	1 fM	[80]
AuAg@Ag hollow cubic NSs	4-MPh	–	1 aM	[81]
Ag-Au@NF	Rhodamine 6G	–	0.1 nM	[82]
AuNP@PDOP@AgNP	MB	3.5 × 10^5^	–	[83]
Xylan-capped Au@Ag	Sudan I	–	1 nM	[84]
Xylan-capped Au@Ag	4-MBA	1.24 × 10^8^	1 nM	[84]
Au@AgNPs	4-MPY	3.5 × 10^7^	<1 nM	[85]

**Table 3:** SERS performances of metal-ZnO-based nanostructures for biological/chemical sensing (AgNPs = Ag nanoparticles; TMPyP = (meso-tetra(N-methyl-4-pyridyl)porphine tetrachloride; WGM = whispering gallery mode).

SERS Substrates	Detected Molecules	EF or AEF	LOD	References
ZnO Nanowires with AgNPs	TMPyP	–	100 nM	[86]
ZnO heterostructure with AgNPs	Malachite green	–	0.1 pM	[87]
Ag/ZnO heterostructure	Methylene orange	1.3 × 10^10^	1 pM	[88]
ZnO/graphene/Ag WGM microcavity	Rhodamine 6G	9.5 × 10^11^	1 fM	[89]
Hollow ZnO@Ag nanospheres	Nitrite	–	3 nM	[90]
Ag/ZnO nanorods	Pioglitazone	–	1 nM	[91]
Ag/ZnO nanorods	Phenformin	–	5 nM	[91]
Ag/ZnO/Au nanorods	λ-DNA	–	0.3 nM	[92]
Au/ZnO nanorods	Dopamine	1.2 × 10^4^	–	[93]
ZnO nanorods with AuNPs	Methylene blue	–	1 nM	[94]
Au/ZnO hollow urchins	Adenine	–	1 μM	[95]
Au/ZnO hollow urchins	Thiophenol	–	10 nM	[95]

**Table 4:** SERS performances of metal-oxide nanostructures for biological/chemical sensing (vdW MoO_3_ = van der Waals molybdenum trioxide; TMOs = transition metal oxides; W_18_O_49_ = WO_2.72_ = non-stoichiometric tungsten oxide; WO_3−*x*_ = non-stoichiometric tungsten oxide; QD = quantum dot; NW = nanowire; ZrO_2_ = zirconia; CoFe_2_O_4_ = cobalt ferrite).

SERS Substrates	Detected Molecules	EF or AEF	LOD	References
Few-layered vdW MoO_3_ nanosheets	Rhodamine 6G	–	20 nM	[96]
TMOs planar SERS chips	Rhodamine 6G	–	1 nM	[97]
W_18_O_49_-H_2_ nanowire film	Rhodamine B	4.4 × 10^5^	0.1 μM	[98]
WO_3−*x*_-based SERS substrate	Rhodamine B	1.2 × 10^6^	0.1 μM	[99]
WO_3−*x*_QD@AgNW	Methylene blue	–	1 μM	[100]
ZrO_2_@CoFe_2_O_4_@Au nanoparticles	Thiolated malachite green	5 × 10^10^	–	[101]
